# Tumor-derived biomimetic nanozyme with immune evasion ability for synergistically enhanced low dose radiotherapy

**DOI:** 10.1186/s12951-021-01182-y

**Published:** 2021-12-28

**Authors:** Chunyu Huang, Zeming Liu, Mingzhu Chen, Liang Du, Chunping Liu, Shuntao Wang, Yongfa Zheng, Wei Liu

**Affiliations:** 1 Key Laboratory of Artificial Micro- and Nano-Structures of Ministry of Education, School of Physics and Technology, Wuhan University, Wuhan, 430072 People’s Republic of China; 2 Department of Oncology, Renmin Hospital of Wuhan University, Wuhan, 430060 Hubei China; 3 Department of Plastic and Cosmetic Surgery, Tongji Hospital, Tongji Medical College, Huazhong University of Science and Technology, Wuhan, 430030 China; 4 Department of Breast and Thyroid Surgery, Union Hospital, Tongji Medical College, Huazhong University of Science and Technology, Wuhan, 430022 China

**Keywords:** Low-dose radiotherapy, Tumor-derived exosomes, Pyrite nanozyme, Mitochondrial damage, GSH depletion

## Abstract

High doses of radiation can cause serious side effects and efficient radiosensitizers are urgently needed. To overcome this problem, we developed a biomimetic nanozyme system (CF) by coating pyrite (FeS_2_) into tumor-derived exosomes for enhanced low-dose radiotherapy (RT). CF system give FeS_2_ with immune escape and homologous targeting abilities. After administration, CF with both glutathione oxidase (GSH-OXD) and peroxidase (POD) activities can significantly lower the content of GSH in tumor tissues and catalyze intracellular hydrogen peroxide (H_2_O_2_) to produce a large amount of ·OH for intracellular redox homeostasis disruption and mitochondria destruction, thus reducing RT resistance. Experiments in vivo and in vitro showed that combining CF with RT (2 Gy) can provide a substantial suppression of tumor proliferation. This is the first attempt to use exosomes bionic FeS_2_ nanozyme for realizing low-dose RT, which broaden the prospects of nanozymes.

**Supplementary Information:**

The online version contains supplementary material available at 10.1186/s12951-021-01182-y.

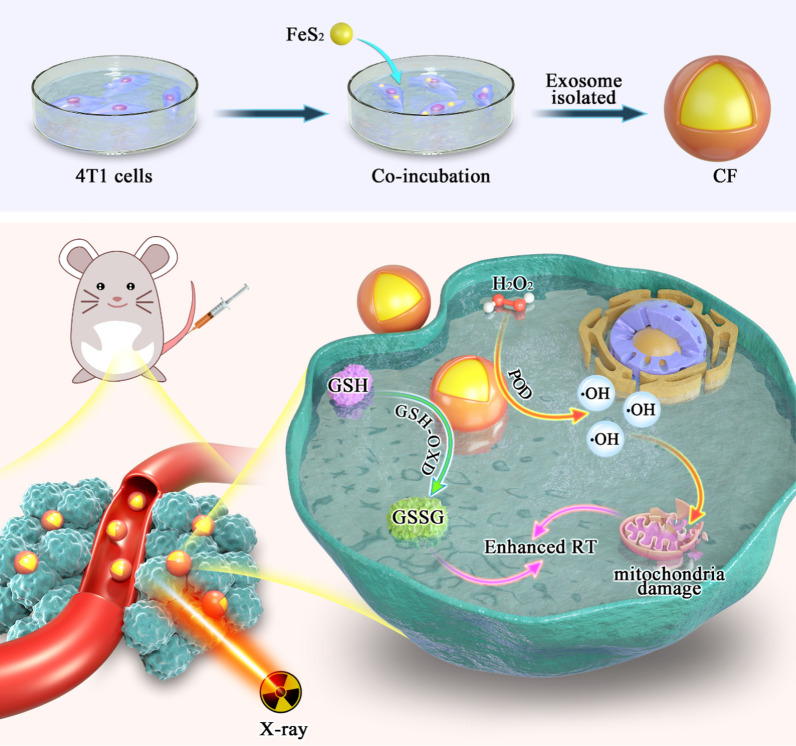

## Introduction

Despite advances in scientific study, cancer, which can strike at any age, remains a severe threat to human life and health in today’s society [1–3]. Radiotherapy (RT) is used to keep almost half of all cancer patients alive, either alone or in combination with other innovative treatments [4–6]. From a mechanical standpoint, RT relies on the use of high-energy X-rays or gamma rays to cause radiation-induced DNA damage or to stimulate the formation of large amounts of toxic reactive oxygen species (ROS), of which about 90% are produced by mitochondria [7]. Cell apoptosis occurs when the rate of ROS synthesis exceeds the cell’s ability to neutralize these free radicals [8]. This allows tumor cells to be killed and the tumor to be abated. Long-term exposure to higher doses of radiation, on the other hand, can induce a variety of negative side effects, including fatigue, nausea and some digestive system diseases, etc. [9]. Furthermore, the tumor microenvironment (TME) in solid tumors exhibits high levels of GSH expression, which can inhibit anti-tumor RT [10, 11]. Moreover, because GSH is a reducing agent, it can directly eliminate ROS which reduces the effectiveness of ROS-based therapies [12]. Therefore, reducing GSH level can effectively improve RT efficacy.GSH elimination can be accomplished through a variety of pathways due to the various pathways of glutathione metabolism and the various types of chemical reactions involving glutathione, and converting glutathione to an oxidation state through direct interactions has become one of the most commonly used methods for reducing glutathione levels [12–14]. Bao et al. for example, created a composite nanomaterial containing MnO_2_ to improve tumor hypoxia, alleviate and lower intracellular GSH levels, and therefore achieve radiation sensitization [15]. Qu et al. developed MoS_2_@AIBI-PCM, a composite nanomaterial in which MoS_2_ can efficiently produce GSH oxidation without releasing hazardous metal ions, resulting in significant tumor death and good biocompatibility during therapy [16]. As a novel nano-materials, FeS_2_ nanozyme was verified to obtain both glutathione oxidase (GSH-OXD) and high-activity peroxidase (POD) [17]. Nanozymes have great advantages over natural enzymes [18–20]. FeS_2_ nanozyme can reduce GSH and catalyze H_2_O_2_ to produce sufficient ·OH to destroy mitochondria, which is expected to achieve a good synergistic effect combined with radiotherapy [21]. Despite the fact that these elements have proven effective against the GSH system, they are hardly to reach the tumor site [22]. These nanomaterials have a low immune escape capacity and are easily cleared by liver and kidney organs in the bloodstream; thus, their targeting ability and anti-tumor efficiency are considerably reduced [23, 24].

Cancer cell-derived exosomes (CDE) are endogenous vesicles ranging in size from 50 to 200 nm that are recovered from multivesicles or retrieved by incubating cell supernatants with appropriate separation kits [25–27]. Because they may be generated from tumor cells and are less likely to elicit a clearance response than manufactured drug delivery platforms, such vesicles, given by cancer cells themselves, provide improved drug delivery prospects [28–30]. Furthermore, exosomes have a high level of non-immunogenicity, making them resistant to phagocytosis by macrophages [31]. Exosomes are also simpler to penetrate from blood arteries to tumor tissues for precise medication administration or nanomaterial delivery [32]. Therefore, it stimulates us to coat nanozymes with CDE to overcome tumor RT resistance.

**Scheme 1:**
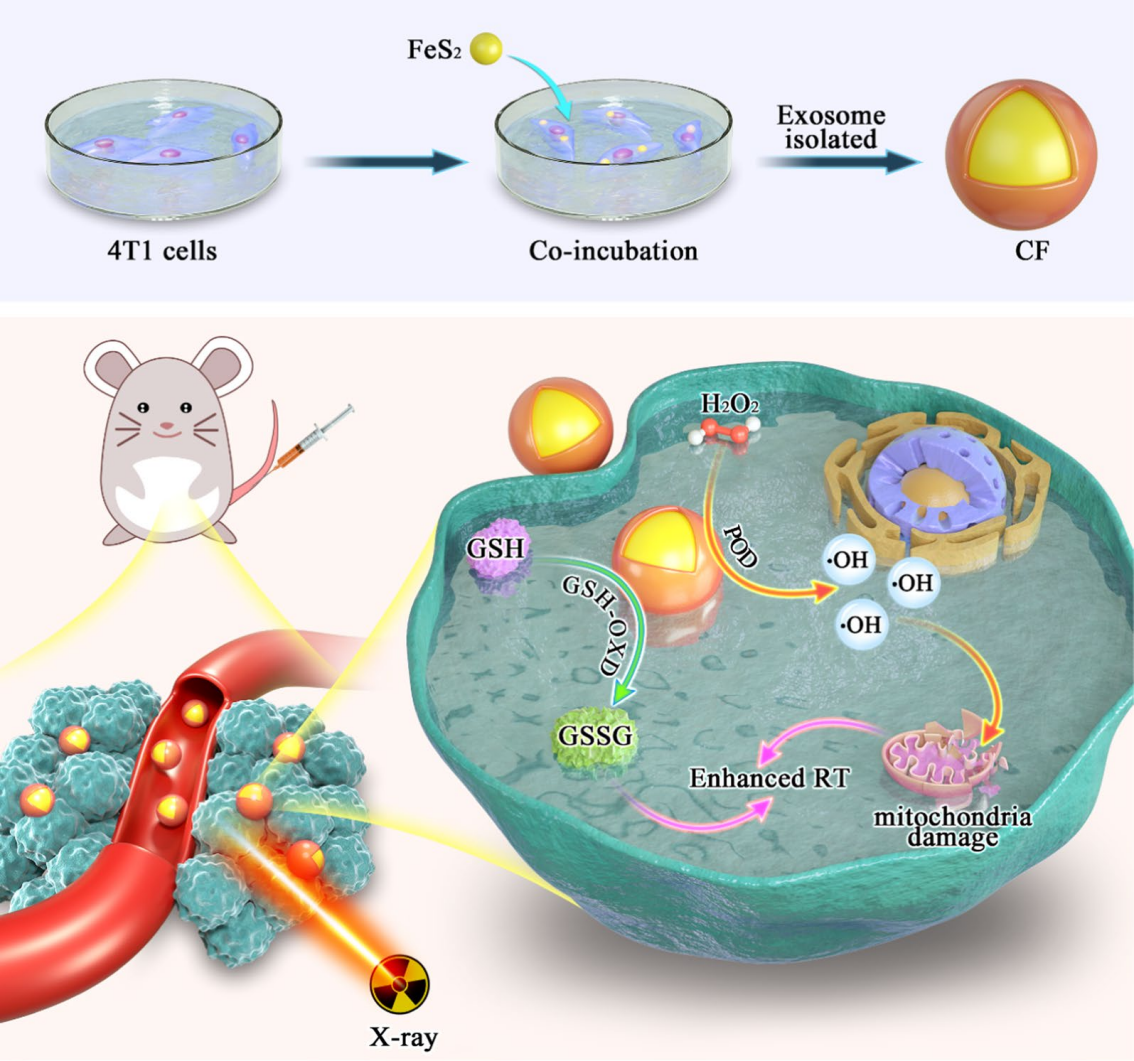
Schematic illustration of tumor-derived biomimetic nanozyme with immune evasion ability for synergistically enhanced low dose radiotherapy

The combined application of FeS_2_ nanozyme and CDE to augment low-dose radiation was originally reported in this work. By coating FeS_2_ with CDE, a composite CF system was created (Scheme 1). Because CF has both homologous targeting and dual enzyme properties, the exosome membrane can enhance FeS_2_ blood circulation time in vivo, and simultaneously help FeS_2_ actively targeting to tumor tissues. Subsequently, FeS_2_ nanozyme with GSH-OXD and POD activity can lower intracellular GSH levels. It catalyzes hydrogen peroxide in tumor cells to generate ·OH, which disrupts redox equilibrium and kills mitochondria, resulting in radiation sensitization. It’s important to mention that in the therapeutic process, we can get a powerful radiation sensitization with only 2 Gy of RT synergistic with CF, which is better than 6 Gy RT. CF system offers higher biological potential uses in the clinical. Finally, our findings broaden the use of exosomes and provide fresh insights into the development of exosom-based oncology therapeutic systems.

**Fig. 1:**
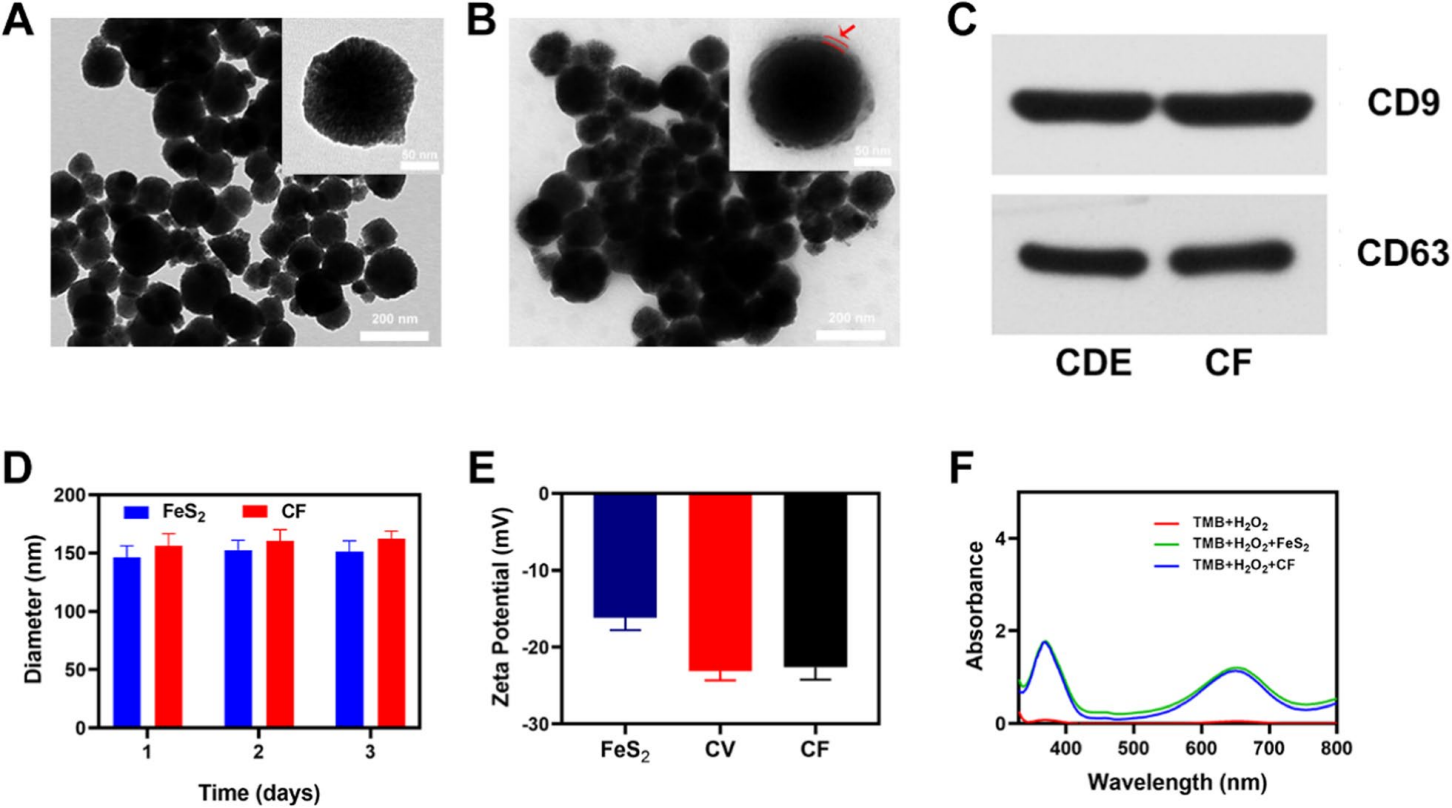
TEM image of FeS_2_ (**A**) and CF (**B**) with the inset image showing the single image of corresponding materials. **C** Western blotting was used to measure the EXO markers CD9 and CD63. **D** Statistical graph of the measured diameter of FeS_2_ and CF. **E** Zeta potential values for FeS_2_, CV, and CF nanovesicles. **F** UV − vis absorbance spectra and color changes of TMB in different reaction systems

In this study, 4T1 cells were co-incubated with FeS_2_ materials, and exosome separation kits were used to obtain exocytosed CF, which was then employed to build a CF system. Transmission electron microscopy (TEM) was used to confirm the successful production of CF material. Compared with pure FeS_2_, CF was coated with a layer of exosome membrane with a small thickness (Fig. 1A, B), and this conclusion was further verified by western blotting assay (Fig. 1C). On the CDE membrane, the exosome protein markers CD63 and CD9 were identified [21, 28]. On CF loaded with FeS_2_, the CD63 marker and another exosome marker (CD9) were also found, showing that the exosome membrane proteins on CF were not disturbed. Exosome membranes offer a lot of potential in the field of material delivery, and they’re a lot better than standard drug delivery systems like erythrocyte membranes that don’t have any targeting and don’t improve the amount of material that gets into tumor tissues [33]. In addition, during the three days of pure FeS_2_ and CF stored in the phosphate buffer solution (PBS) environment (4 ℃), their particle sizes almost did not change significantly. During the 3 days, the particle sizes of FeS_2_ were 146.2 ± 1.6 nm, 152.6 ± 1.8 nm, and 151.0 ± 1.4 nm, respectively, while the particle sizes of CF were 153 ± 2.1 nm, 159.4 ± 2.5 nm, and 157.5 ± 1.9 nm (Fig. 1D), which also indicates that both FeS_2_ and hybrid CF have good stability and can be used in subsequent biological experiments. This quality is unquestionably critical, as many materials have good properties, but their instability will prevent them from being used [34]. The Zeta potential of different particles was shown in Fig. 1E, with cancer cell vesicle (CV) and CF having zeta potentials of roughly about − 23.1 and − 22.6, respectively. We investigated POD-like activity of FeS_2_ and CF based on the oxidation of 3,3′,5,5′-tetramethylbenzidine (TMB) in the presence of H_2_O_2_. The results show that both pure FeS_2_ and FeS_2_ encapsulated by exosome membrane can catalyze H_2_O_2_ to produce ·OH (Fig. 1F). These findings show that CF has an exosomal membrane structure and that it will be endocytosed into 4T1 tumor cells via extracellular action to alter the tumor microenvironment.

**Fig. 2:**
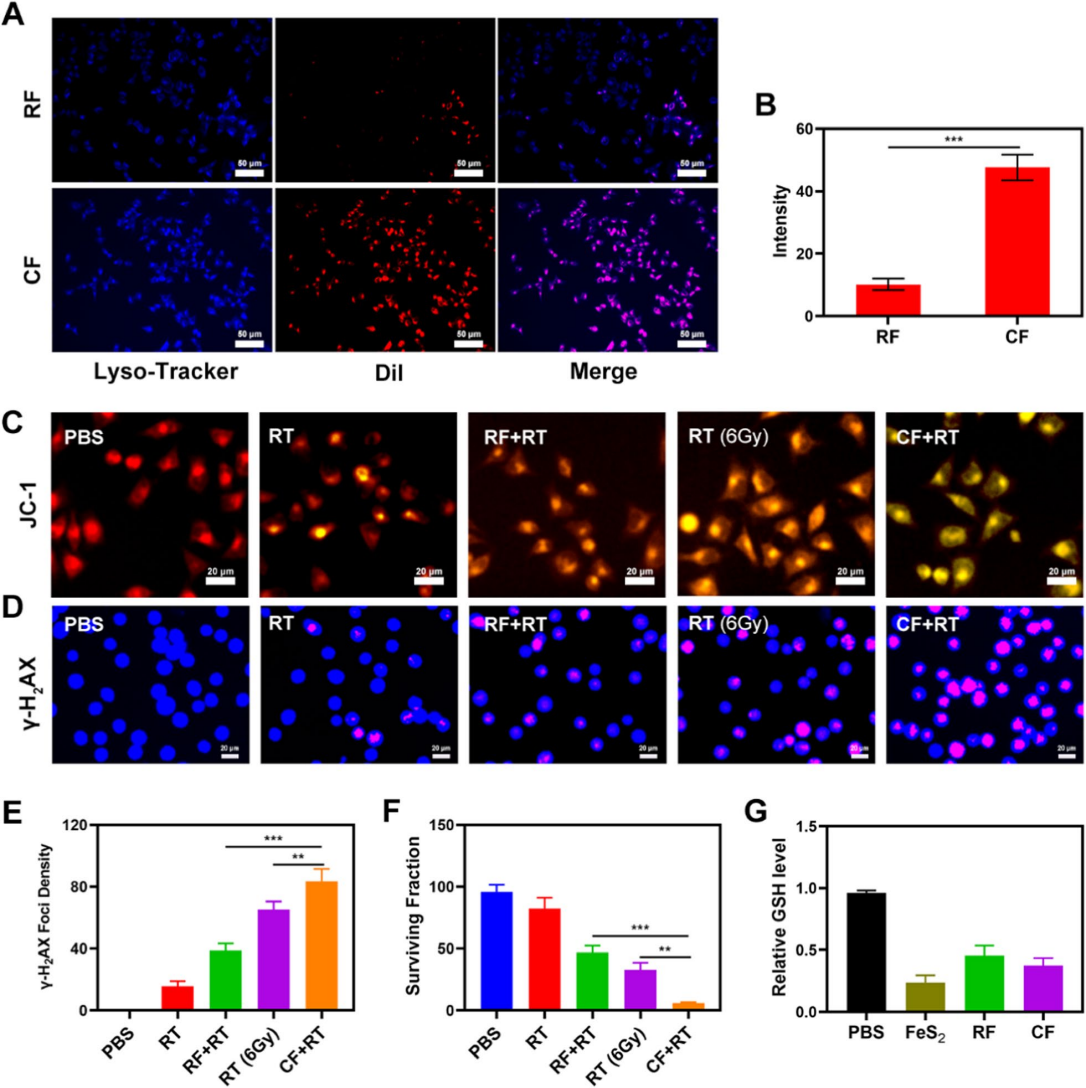
Co-localization of Lyso-Tracker Green FM (blue) and Dil (red) for RF and CF over time in 4T1 tumor cells. **B** Dil fluorescence intensity of **A** determined using ImageJ software. **C** JC-1 (green) for JC-1 monomer and red for JC-1 aggregate fluorescence image under different treatment. **D** Nuclear condensation and DNA fragmentation were visualized using DAPI and γ-H_2_AX staining in cells treated as indicated, with representative pictures presented. **E** The density of γ-H_2_AX foci was determined based on analyses of 100 cells per treatment group (γ-H_2_AX foci/100 μm^2^, n  = 3). **F** Colony formation assays were conducted using 4T1 cells treated with 2 or 6 Gy of radiation (n = 3). **G** The impact of FeS_2_ on the intracellular level of GSH was estimated using a GSH assay kit (n  = 5). Significant differences among groups as calculated using the student’s t test. **P  < 0.01, ***P  < 0.005

The CF system is well-structured and characterized in terms of performance. In vitro anti-tumor trials are currently underway. Although FeS_2_ can alter the ecological balance of the cancer cells and so enhance the efficacy of radiation, it can only do so when it is present in the tumor tissue. The immune system may recognize diverse foreign invaders’ stimuli, and a portion of that stimulation can activate the immune response, resulting in immunity, while preventing other stimuli, resulting in tolerance [35]. Due to homology, which is connected to exosome membrane proteins, the immune system does not attack the exosomes released by cancer cells. Nanomaterial-coated exosomes can be directed to tumor cells, recognized by cancer cells, and engorged to release nano-material, whereas other drug delivery systems that do not have targeting capabilities. This internalization effect can be tracked by staining CF and red blood cell membranes coated FeS_2_ (RF) nanovesicles with Dil dye and co-incubation with 4T1 cells and staining with commercial lysosome Lyso-Tracker Green probe to verify the ability of CF to be internalized by tumor cells in vitro (co-localization assay). For comparison, red cell membranes were coated with FeS_2_ to form RF. After 2 h incubation, it was clear that a significant quantity of CF had been endocytosed, whereas RF only had a partial Dil fluorescent impact (Fig. 2A, B), demonstrating that exosomes might be employed as a perfect carrier of FeS_2_ to target tumor tissues. As shown in Additional file 1: Figure S2, hemolysis assay verified that CF is stable in blood, indicating good biocompatibility of CF. In order to demonstrated the universality of exosome systems, we then prepared manganese dioxide nanozyme (MnO_2_), as shown in Additional file 1: Figure S3, and used CDE to coat MnO_2_ (CM) (Additional file 1: Figure S4). Furthermore, during the several days of MnO_2_ and MF stored in the PBS environment (4 ℃), the diameter of MnO_2_ and CM has barely changed (Additional file 1: Figure S5). MnO_2_ has good catalase activity (CAT), which can catalyze H_2_O_2_ into O_2_. CM also has good CAT activity, indicating that exosome membrane does not affect the enzyme activity (Additional file 1: Figure S6). Even at high concentrations, the number of MnO_2_ swallowed by 4T1 cells was low (Additional file 1: Figure S8). As MnO_2_ can achieve radiotherapy sensitization by alleviating tumor hypoxic microenvironment, while CM can actively target cells, as shown in Additional file 1: Figure S7, the colony formation assays also proves that CM has better synergetic cell killing effect compared with MnO_2_. Then, the changes of mitochondrial membrane potential (MMP) in tumor cells were monitored by JC-1 (5,5′,6,6′-tetrachloro-1,1′,3,3′-tetraethyl-imidacarbocyanine) probe method. The JC dye normally builds up in the mitochondria, where it clumps together to give a red fluorescence. When mitochondria are injured and MMP levels are low, however, the JC monomer is released into the cytoplasm, resulting in green fluorescence [36]. Figure 2C demonstrates that cells treated with FeS_2_  +  RT had a high green/red fluorescence ratio, which is consistent with the reduced mitochondrial damage generated by FeS_2_. FeS_2_ will enter TME with twofold enzyme activity once CF is endocytosed by tumor cells, reducing GSH content while also catalyzing enough H_2_O_2_ to generate ·OH in situ, causing significant mitochondrial damage. Radiotherapy ought to be more sensitive as a result of mitochondrial injury. Double-stranded DNA breaks (DSB) in tumor cells when exposed to radiation, which provides insights into the radiation sensitization, and measuring the fluorescence intensity of γ-H2AX, is a good and intelligent way to verify the DSB formation after cell damage [37]. Therefore, we detected H2AX foci in the nucleus after treatment in different groups. After 2 Gy of radiotherapy, there was substantial DNA damage, and the DSB effect increased as the dose was increased. It's important to mention that 2 Gy radiation mixed with RF only got about 40% γ-H2AX formation, but 2 Gy paired with CF got up to 76.8% γ-H2AX foci development. The synergistic effect of the targeting ability of the exosome membrane, the dual nano enzyme activity of FeS_2_, and radiation sensitization was linked to the uniform and substantial difference between each experimental group. Furthermore, Colony formation assays revealed that the control group’s cell viability was largely unaltered, whereas the RT with RF group had moderate tumor growth inhibition (Fig. 2F). CF  +  RT system had the best tumor growth inhibition rate (90%), there are significant differences compared with each other experiment group, indicating that CF mediated improved ·OH content of TME can effectively exert influence on mitochondria and thus enhance the RT effect to realize tumor growth inhibition. Figure 2G also verifies that our FeS_2_ has a good effect on GSH consumption. Together, these results drive our continued exploration of anti-tumor efficacy in vivo.

**Fig. 3:**
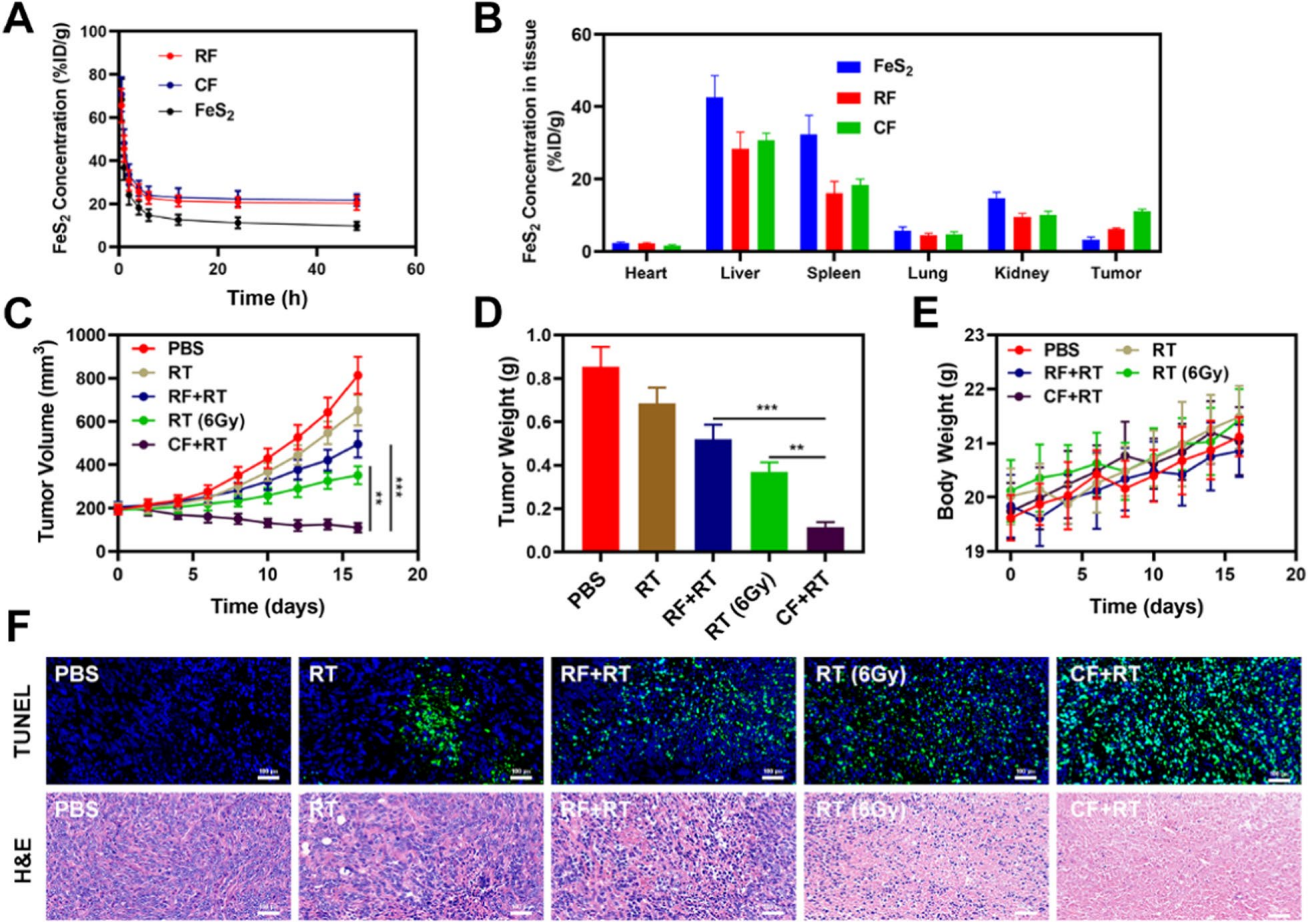
**A** Pharmacokinetic behavior of FeS_2_, RF, and CF in mice following i.v. administration at doses of 2.5 mg Fe/kg. Data are presented as mean  ±  SD (n  = 3). **B** Quantitative analysis of FeS_2_ biodistribution in tissues and tumors of tumor-bearing mice injected with FeS_2_, RF, or CF at FeS_2_ dose of 2.5 mg Fe/kg, respectively. **C** Change in tumor-volume curves of 4T1 tumor-bearing mice after treatments. **D** Changes in tumor weight following treatment. **E** The body weight of 4T1 tumor-bearing mice was measured every 2 days after therapy. **F** Following therapy, TUNEL and H&E-stained tumor slice photos of mice are shown. Significant differences among groups as calculated using the student’s t test. **P  < 0.01, ***P  < 0.005

**Fig. 4:**
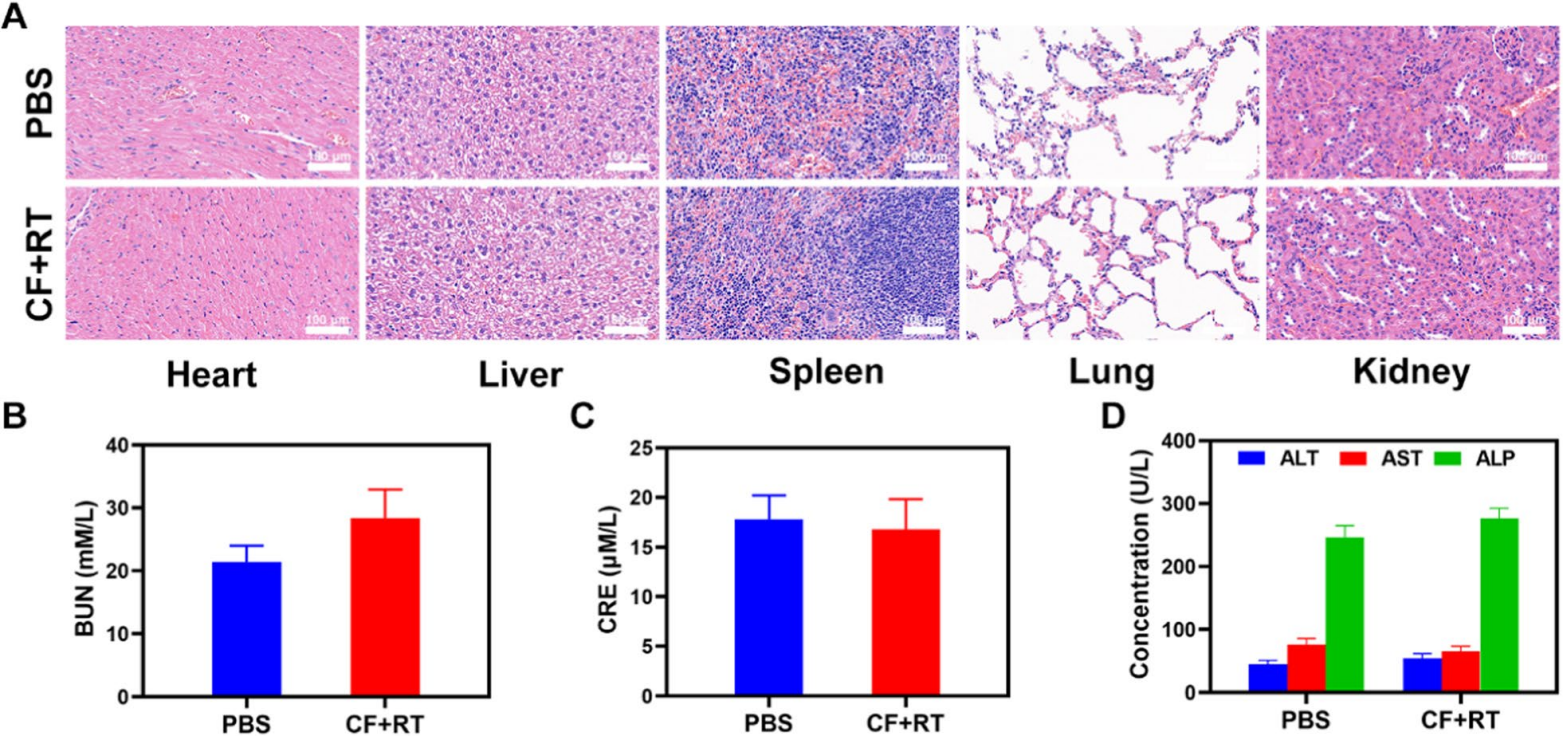
Result of in vivo safety experiments. **A** Histopathological analysis results (H&E) stained images of the major organs, heart, lung, liver, kidneys, and spleen, of mice that were exposed to different treatments 16 days post-injection under laser irradiation. Blood biochemistry data including kidney function markers: **B** liver function markers: BUN, **C** CRE, and **D** ALT, ALP, and AST after various treatments

Given that in vitro testing of the tumor-killing action revealed significant potential. We investigate how CF effects can be amplified in vivo. As a result, we conducted in vivo pharmacokinetic studies to see how exosome membranes affect blood retention. Mice were given FeS_2_, RF, or CF at a dose of 2.5 mg Fe/kg intravenously (Fig. 3A). Both RF and CF demonstrated a greater effect on blood retention when compared to the pure FeS_2_ group. Although the erythrocyte membrane coating protected from the attack of the immune system, it lacked tumor targeting, and the accumulation of FeS_2_ in tumor tissues was only slightly increased, with the CF group having the most visible tumor accumulation. Following that, we investigated the biological dispersion (Fig. 3B). After 12 h of administration, the FeS_2_ mainly accumulated in the liver and spleen of the FeS_2_ group of mice, whereas the CF group demonstrated good tumor targeting and little organ accumulation, demonstrating the exosome membrane’s targeting capabilities. In order to verify the targeting ability of drugs-loaded exosomes, we conducted in vivo pharmacokinetic studies using DOX loaded erythrocyte exosomes (RBC-EXO@DOX) and CDE (CDE-EXO@DOX), respectively. Simultaneously, DOX loaded CF was prepared (CF@DOX). As shown in Additional file 1: Figures S9, S10, RBC-EXO@DOX and CDE both have long circulation. However, RBC-EXO@DOX accumulates less in tumor tissues compared with CDE-EXO@DOX and CF@DOX (Additional file 1: Figure S10). This result shows that tumor-derived exosomes have good tumor targeting. Following that, we looked at the efficacy of CF-mediated anti-tumor therapy in mice with 4T1 tumors. BALB/c mice were subcutaneously injected with 1 × 10^6^ 4T1 cells into the right flank to determine the primary effect of the CF. When the primary tumor volume reached 200 mm^3^, the mice were divided into groups and treated. Tumor-bearing mice were divided randomly into 5 groups (each group included 5 mice): (1) control (PBS); (2) radiotherapy (RT, 2 Gy); (3) RF  +  RT; (4) high dose RT (6 Gy); (5) CF  +  RT. The FeS_2_ concentration was 5 mg/kg in groups 3, and 5. For 16 days, the treatment was given every 4 days. The tumor volumes of the control and RT treated groups increased rapidly during the 2 weeks of treatment, as shown in Fig. 3C. The RF  +  RT group likewise had a nearly moderate tumor-suppressive effect. When these nanomaterials are injected into the caudal vein, when the RF circulates to the tumor tissue and is endocytosed by tumor cells, FeS_2_ is released to the TME, playing the equivalent therapeutic impact. The CF  +  RT system, which included both FeS_2_, had the most potent therapeutic impact, with tumor volume growth curves nearly totally suppressed during therapy. The tumor mass in mice was also consistent with the volume curve (Fig. 3D). No weight changes were detected in the treatment group during this study, indicating that the treatment did not cause any significant systemic toxicity (Fig. 3E), which is significant because many treatments are associated with extremely systemic toxicity, which is extremely detrimental to the future clinical application of the material. We collected tumor tissue slices for staining. TUNEL and H&E staining (Fig. 3F) indicated the presence of a high level of cell necrosis in the CF combined RT therapy group. Furthermore, as shown in Fig. 4, after the treatment of mice's vital organs (heart, liver, spleen, lungs, and kidney) without any inflammation and damage in the body, liver, and kidney indices were also normal. Many nanomaterials have high therapeutic efficacy, however, they are also associated with systemic toxicity, limiting their future clinical applications. The in vivo results show that our innovative combined therapy not only achieves an excellent therapy with biological safety but also enhances tumor ·OH content and reinforces the effect of RT with substantial CF-enhanced therapy.

## Conclusion

Finally, we developed a unique oxidative stress destroyer to enable better radiotherapy. The CF can decrease the content of GSH in the tumor microenvironment and catalyze H_2_O_2_ to produce a large amount of ·OH, the exosome membrane can render FeS_2_ with admirable homologous targeting ability, and all of these elements have an enhanced ability to low-dose RT. Both the in vitro and in vivo studies suggest that our method has a good tumor inhibition effect. Importantly, during treatment, our prepared system showed no evident side effects. In the future, we will continue to investigate the biological applications of the combination of FeS_2_ and other unique vehicles, as well as refine our treatment strategy by incorporating additional nanotechnology and new technologies.

## Supplementary Information

**Additional file 1: Figure S1.** Nanoparticle uptake by RAW 264.7 cells at different incubated concentration (FeS_2_ dose of 25, 50 and 100 μg/mL). Data are presented as mean ± SD (n = 3). **Figure S2.** Hemolysis ratio of CF at different FeS_2_ concentrations. **Figure S3.**TEM image of MnO_2_. **Figure S4.**TEM image of CM. **Figure S5.** Statistical graph of the measured diameter of MnO_2_ and CM. Data are presented as mean ± SD (n = 3). **Figure S6.** Oxygen generation under different conditions measured using a dissolved oxygen meter. **Figure S7.** Nanoparticles uptake by 4T1 cells at different concentration. Data are presented as mean ± SD (n = 3). **Figure S8.** Colony formation assays were conducted using 4T1 cells with different treatment (n = 3). **Figure S9.** Pharmacokinetic behavior of RBC-EXO@DOX, CDE-EXO@DOX, and CF@DOX in mice following i.v. administration. Data are presented as mean ± SD (n = 3). **Figure S10.** Quantitative analysis of DOX biodistribution in tissues and tumors of tumor-bearing mice injected with different formulations. Data are presented as mean ± SD (n = 3).

## Data Availability

All the original data are available upon reasonable request for correspondence authors.
