# Supplementary material for: Tumor-derived biomimetic nanozyme with immune evasion ability for synergistically enhanced low dose radiotherapy
Source: J Nanobiotechnology. 2021 Dec 28;19:457. doi: 10.1186/s12951-021-01182-y (PMC8715603; doi:10.1186/s12951-021-01182-y)
Supplement: Supplementary file 1 — Additional file 1: Figure S1. Nanoparticle uptake by RAW 264.7 cells at different incubated concentration (FeS2 dose of 25, 50 and 100 μg/mL). Data are presented as mean ± SD (n = 3). Figure S2. Hemolysis ratio of CF at different FeS2 concentrations. Figure S3.TEM image of MnO2. Figure S4.TEM image of CM. Figure S5. Statistical graph of the measured diameter of MnO2 and CM. Data are presented as mean ± SD (n = 3). Figure S6. Oxygen generation under different conditions measured using a dissolved oxygen meter. Figure S7. Nanoparticles uptake by 4T1 cells at different concentration. Data are presented as mean ± SD (n = 3). Figure S8. Colony formation assays were conducted using 4T1 cells with different treatment (n = 3). Figure S9. Pharmacokinetic behavior of RBC-EXO@DOX, CDE-EXO@DOX, and CF@DOX in mice following i.v. administration. Data are presented as mean ± SD (n = 3). Figure S10. Quantitative analysis of DOX biodistribution in tissues and tumors of tumor-bearing mice injected with different formulations. Data are presented as mean ± SD (n = 3). [file 12951_2021_1182_MOESM1_ESM.docx]

**Experimental section**

**Materials and reagents.**

Deionized (DI) water was obtained by an 18 MΩ cm (SHRO-plus DI) system. Phosphate buffer solution (PBS) and bovine serum albumin (BSA) were purchased from Thermo-Fisher (USA). Polyvinyl pyrrolidone (MW: 10 000), iron (III) chloride hexahydrate (FeCl_3_·6H_2_O, 99%), ethylene glycol (EG), anhydrous sodium acetate (NaAc, 99%), Paraformaldehyde (PFA), 1, 1’-dioctadecyl-3, 3, 3’, 3’-tetramethylindocarbocyanine perchlorates (Dil), and cell counting kit-8 (CCK-8) were obtained from Sigma-Aldrich (USA). Sulfur powder (S, 99%) was obtained from Shanghai Rongtai Pharmatech Co., Ltd. JC-1 Mitochondrial Membrane Potential Assay Kit was purchased from Yeasen Biotechnology (Shanghai) Co., Ltd. The γ-H_2_AX antibody was purchased from Abcam Company. The other reagents used in this work were purchased from Sinopharm Chemical Reagent (China) and Aladdin-Reagent (China).

**Preparation of** **pyrite nanozymes (FeS_2_).**

The pyrite nanozymes with a size of 151 nm were prepared by a one-pot solvothermal method([1](#_ENREF_1)). In a typical synthesis, 0.7 g of PVP was dispersed into 30 mL of EG solution, and then 0.5 g of FeCl_3_·6H_2_O was added under

constant magnetic stirring. Next, 3.6 g of NaAc was added into above mixture under vigorous stirring. After that, 0.4 g of S powder was added, and the resultant mixture was ultrasonicated for 1 h to form a homogeneous dispersion. Then, the reaction mixture was transferred into a 40 mL Teflon-lined stainless-steel autoclave, which was sealed and maintained at 473 K for 12 h. After the reaction was cooled to room temperature naturally, the resultant black precipitates were collected by centrifugation at 10 000 rpm for 10 min. Then, the collected black products were washed with CHCl_3_ to remove the excess S and using absolute alcohol and ultrapure water several times to remove impurities. After centrifugation, the products were dried in a vacuum lyophilizer overnight for further characterization.

**Preparation of cancer exosome (CDE) biomimetic FeS_2_ nanoparticles (CF).**

The pure CDE were prepared according to the standard protocol of Exosome Isolation Reagent (RIBOBIO biotechnology co. LTD, China) ([2](#_ENREF_2)). For CF preparation, 4T1 cancer cells were cultured for 3 days and then the growth medium was rinsed out, replaced with fresh medium containing 1mg FeS_2_ and incubated for 2 days at 37 °C and 5% CO_2_ atmosphere. Then the medium was replaced by the fresh medium without FBS and the cells were incubated at 37 °C and 5% CO_2_ atmosphere for another 2 days. Then all the supernatant was collected and centrifuged at 1125 g for 5 min to eliminate the cells and debris. The CF in supernatant isolated by centrifuged at 10000 rpm for 5 min. CF was stored at 4℃ for further use.

**Preparation of erythrocyte membranes biomimetic FeS_2_ nanoparticles (RF).**

The red blood cell-vesicles were obtained by using low-osmosis method([3](#_ENREF_3)). The RF were obtained by using an extrusion method([4](#_ENREF_4)). Briefly, 0.1 mg of FeS_2_ were mixed with 1 mg of red blood cell-vesicles, which were quantified by lyophilization and extruded through 100 nm polycarbonate membranes. The RF were stored in PBS at 4 °C for the following experiments.

**Synthesis of hollow MnO_2_ nanoparticles (MnO_2_).**

Solid silica nanoparticles (sSiO_2_) were synthesized following the reported method.1 Then an aqueous solution of KMnO_4_ (300 mg) was dropwise added into the suspension of sSiO2 (40 mg) under ultrasonication. After 6 h, the precipitate was obtained by centrifugation at 14,800 rpm. The asprepared mesoporous MnO_2_-coated sSiO_2_ was dissolved in 2M Na_2_CO_3_ aqueous solution at 60 °C for 12 h. The obtained hollow mesoporous MnO_2_ nanoparticles (MnO_2_) were centrifuged and washed with water several times.

**Preparation of CM**

4T1 cells were treated with MnO_2_ (200 μg/mL) for 6 h in 7.5 cm dishes. Then the media were discarded and replaced with fresh one without MnO_2_. After 16 h incubation, the debris was discarded at 1,000 rpm for 15 min and then the supernatants were further centrifuged at 8,000 rpm for 15 min to pellet out CM. Then, the obtained pellets were washed with PBS and resuspended in PBS for further experiments.

**Preparation and characterization of DOX loaded exosomes (CDE-EXO@DOX) or DOX loaded red blood cell exosomes (RBC-EXO@DOX)**

The exsomes (EXO) derived from 4T1 cancer cells or red blood cells were prepared according to the standard protocol of Exosome Isolation Reagent (RIBOBIO biotechnology co. LTD, China). To load DOX into the exosomes, the different exosomes (0.5 mg) and DOX (100 μg in 10 μL DMSO) were mixed in 250 μL PBS in 0.4 cm cuvette (Bio-Rad). Electroporation was then carried out at 250 V and 350 μF on a Bio-Rad Gene Pulser Xcell Electroporation System. After electroporation, the mixture was incubated at 37 °C for 30 min to allow the recovery of the membrane of the electroporated exosomes. The CDE-EXO@DOX and RBC-EXO@DOX were collected by using Exosome Isolation Reagent again and stored at 4 ℃ for further use.

**Preparation of DOX loaded CF (CF@DOX)**

To load DOX into the CF, the CF (0.5 mg) and DOX (50 μg in 10 μL DMSO) were mixed in 250 μL PBS in 0.4 cm cuvette (Bio-Rad). Electroporation was then carried out at 250 V and 350 μF on a Bio-Rad Gene Pulser Xcell Electroporation System. After electroporation, the mixture was incubated at 37 °C for 30 min to allow the recovery of the membrane. The CF@DOX were collected by using Exosome Isolation Reagent again and stored at 4 ℃ for further use.

**Characterization of prepared formulations.**

XRD was measured on a D8 Advance (Bruker-AXS, Germany). The morphology of nanoparticles (NPs) was observed by TEM (JEM-2010HT, Japan) at an accelerating voltage of 120 keV. The hydrodynamic diameter and zeta potential of nanoparticles suspended in 1 × PBS or FBS were measured by dynamic light scattering (DLS) (Nano-Zen 3600, Malvern Instruments, and UK).

**Western blotting for the key proteins in CDE and CF**.

The CDE and CF were lysed in RIPA lysis buffer and then subjected to western blot analysis. The primary antibodies used included anti-CD63 (Abcam, ab216130) and anti-CD9 (Santa Cruz, SC-7964). All primary antibodies were diluted to 1:2000.

**POD-like activity of CF**

The POD-like activity of FeS_2_ nanozymes was assessed using TMB as the substrate in the presence of H_2_O_2_. To determine whether FeS_2_ show POD-like activity, 10 μL of FeS_2_ nanozymes or CF (100 mg/mL FeS_2_) was added into 84 μL of 0.2 M HAc−NaAc buffer solution (pH 4.5) containing 1 μL of TMB (20 mg/mL in DMSO) and 5 μL of H_2_O_2_ (10 mM). The UV−vis absorbance spectra of oxidized TMB were recorded via a microplate reader (Tecan, Switzerland).

**Cell lines and animal model.**

4T1 mouse breast cancer cell lines was provided by the College of Life Science of Wuhan University. All the cells were cultured in the standard cell medium recommended by American Type Culture Collection. Female BALC/c mice aged 4-5 weeks were purchased from Vital River Company (Beijing, China). 1×10^6^ 4T1 cells suspended in 100 μL PBS were subcutaneously injected into each mouse to establish the tumor models. After the tumor volume reached around 200 mm^3^, the tumor bearing mice were used for further experiments. The animal experiments were carried out according to the protocol approved by the Ministry of Health in the People’s Republic of PR China and were approved by the Administrative Committee on Animal Research of the Wuhan University.

***In vitro* tumor-specific uptake.**

At first, Dil labeled CF and RF (Containing100 μg/mL FeS_2_) were incubated with 4T1 cells for 2 h at 37 °C. The cells were then washed with PBS several times, fixed with PFA for 30 min at room temperature, stained with Lyso-Tracker Green and then imaged by using a fluorescence microscope (IX81, Olympus, Japan). The fluorescence intensity was measured by IamgeJ software.

***In vitro* Immune Evasion Study**

RAW 264.7 were seeded in 12-well plates and cultured for 12 h. Different concentrations of FeS_2_ and CF (i.e., FeS_2_ dose of 25, 50, and 100 mg/mL) were added the medium, and the cells grown without any particles were used as control. Then the cells were washed three times and then incubated for 4 h at 37 ℃, 5% CO_2_, and then washed with PBS three times. To quantify nanoparticle uptake, 1 mL aqua regia was added to the cells. The mixture was left at room temperature for 12 h, followed by annealing at 70 ℃ for 6 h to remove the acids. The sample was then resuspended with 1 mL DI water and the Fe content in each sample was determined by using an inductively coupled plasma-atomic emission spectrometer (ICP-AES; iris Intrepid II XSP, Thermo Elemental, United States).

**γ-H_2_AX immunofluorescence analysis.**

4T1 cells were seeded in 24-well plates and then cultured for 24 h at 37 °C under hypoxia condition. Then the cells were treated with 1) Control (PBS); 2) Radiotherapy (RT, 2Gy); 3) RF+RT; 4) High dose RT (6Gy); 5) CF+RT. The FeS_2_ concentration was 200 μg/mL in groups 3, and 5. After RT treatment for 2 h, the cells were fixed with 4% paraformaldehyde for 10 min, rinsed with PBS, permeabilized with methanol for 15 min at -20 °C and then rinsed with PBS again. Then the cells were exposed to a blocking buffer (1% bovine serum albumin (BSA) in PBS solution) for 1 h at room temperature and further incubated with anti- phospho-histone γ-H_2_AX mouse monoclonal antibody (dilution 1:500) overnight at 4 °C. After washing with PBS, the cells were incubated with Cy5-conjugated sheep anti-mouse secondary antibody (dilution 1:500) for 1 h at room temperature. Excess antibody was removed by rinsing the coverslips in PBS. Cell nuclei were stained by DAPI for 5 min at room temperature. The cells were imaged via confocal fluorescence microscopy (IX81). Quantitative analysis of γ-H2AX foci density (foci/100 μm^2^) was performed by automatic counting using the ImageJ software for n = 100 cells in each treatment group.

**JC-1 mitochondrial membrane potential assay**

For the JC-1 assay, 4T1 cells were co-incubated with five different groups: 1) Control (PBS); 2) Radiotherapy (RT, 2Gy); 3) RF+RT; 4) High dose RT (6Gy); 5) CF+RT. The cells were stained with JC-1 for 30 min before washing with PBS. Then, the mitochondrial damage/disruption was detected by fluorescence microscopy (IX81).

**GSH depletion activity of CF**

The GSH depletion activity assay was carried out employing DTNB as a probe, which can react with thiol groups (−SH) in GSH to produce a yellow product with maximal absorption at 412 nm. All the experiments need to be examined in the dark because GSH is easily oxidized in light. Specifically, different formulations reacted with GSH solution (0.4 mM) in 0.2 M HAc−NaAc buffer (pH 4.5). Subsequently, the nanoparticles were separated by centrifugation. The supernatants were added with DTNB (0.2 mM) solution to react thoroughly. The mixture was detected for the absorbance of 412 nm.

**Hemolysis assay**

A hemolysis assay was carried out to evaluate the cytotoxicity of CF in vitro. Rabbit heart blood (5 mL) was stabilized by ethylenediamine tetraacetic acid (EDTA) (0.2 mL), an anticoagulant agent. Then red blood cells (RBCs) were obtained from the rabbit heart blood by centrifugation and washing with PBS (2%). 0.5 mL of the RBC solution was then mixed with 0.5 mL CF PBS solution at different concentrations (25, 50 and 100 μg/mL FeS_2_). Water and PBS were used as the positive and negative controls, respectively. All samples were mixed gently and kept at room temperature for 3 h. The absorbance of each supernatant, obtained by centrifugation, was measured at 570 nm on a UV–vis photospectrometer. The hemolysis ratio was calculated by using the formula: hemolysis ratio = (sample absorbance − negative control absorbance) / (positive control absorbance − negative control absorbance) × 100%.

**Evaluation the generation of oxygen water**

MnO_2_ and CM (fixed the concentration of MnO_2_ with 50 μg/mL were suspended in 3% H_2_O_2_ solution (8 mL), respectively. A DOG-3082 oxygen dissolving meter was used to monitor the real-time concentration of oxygen.

***In vitro* cancer targeting study of MnO_2_ and CM.**

4T1 cells were seeded in 24-well plates and cultured for 12 h. Different concentrations of MnO_2_ and CM were added the medium. Then the cells were incubated for 2 h at 37 °C, 5% CO_2_, and then washed with PBS three times. The cells were then fixed with PFA for 30 min at room temperature, stained with Lyso-Tracker Green and then imaged by using a confocal laser scanning microscope (CLSM; IX81, Olympus, Japan). The nanoparticles uptake was measured by ICP-MS as described above.
**Clonogenic assay.**

4T1 cells were seeded in 6-well plates with a different amount (125, 250, 500, 1000 and 2000) per well and incubated at 37 ℃ for 24 h Then the cells were treated with five groups: 1) Control (PBS); 2) Radiotherapy (RT, 2Gy); 3) RF+RT; 4) High dose RT (6Gy); 5) CF+RT. The FeS_2_ concentration was 200 μg/mL in groups 3, and 5. After that, cells were washed with PBS and fresh medium was replaced every three days for 10 days. The colonies were fixed by 4% paraformaldehyde and then stained with Giemsa dye. Only colonies containing at least 50 cells were counted. At last, an evaluation of the effects of different treatments was conducted by counting the survival fraction of the colonies. Each treatment was performed in triplicate. For CM-mediated RT, the cells were treated with five groups: 1) Control (PBS); 2) Radiotherapy (RT, 2Gy); 3) CM; 4) MnO_2_ + RT; 5) CM + RT. The MnO_2_ concentration was 100 μg/mL in groups 3, 4 and 5. Other steps are consistent with the above experiments.

***In vivo* pharmacokinetics and distribution study**

BALB/c mice bearing 4T1 tumor (n = 3) received an intravenous (i.v.) injection of 100 μL PBS containing FeS_2_, RF, or CF at the concentration of 2.5 mg Fe/kg. At various time points after the injection (i.e.,0, 1, 2, 4, 6, 12, 24, and 48 h), 20 μL blood was collected from the tail veins, treated with aqua regia, and then on heated at 70 °C until to obtain clear solutions. And the resultant mixture was left standing still at room temperature for 12 h and then kept in oil bath at 70 °C for 6 h to remove acids, yielding samples for Fe content quantification by using an ICP-AES (iris Intrepid II XSP, Thermo Elemental, USA). To study the biodistribution of particles in various organs, at 12 h after the injection, all mice were euthanized and their hearts, livers, spleens, kidneys, lungs, and tumors were carefully collected, weighted, and finally quantitatively analyzed with ICP-AES as described above. For testing in vivo pharmacokinetics and distribution of RBC-EXO@DOX, CDE-EXO@DOX and CF@DOX, high-performance liquid chromatography (HPLC) was used. The DOX content is 5 mg/kg.

***In vivo* antitumor study.**

1×10^6^ 4T1 cells suspended in 100 μL PBS were subcutaneously injected into each mouse to establish the tumor models. When tumor size reached approximately 200 mm^3^, the mice were divided randomly into 5 groups (each group included 5 mice): 1) Control (PBS); 2) Radiotherapy (RT, 2Gy); 3) RF+RT; 4) High dose RT (6Gy); 5) CF+RT. The FeS_2_ dose was 5 mg/kg in groups 3 and 5. The treatment was conducted every 4 days for 16 days. Mice body weight and tumor volume in all groups were monitored every 2 days. A caliper was employed to measure the tumor length and tumor width and the tumor volume was calculated according to the following formula. Tumor volume = tumor length × tumor width ^2^ / 2. After 16 days of treatment, all the mice were sacrificed. Their blood samples and major organs (i.e., hearts, livers, spleens, lungs, and kidneys) were collected. Three important hepatic indicators (i.e., ALT: alanine aminotransferase, AST: aspartate aminotransferase, and ALP: alkaline phosphatase) and two indicators for kidney functions (i.e., BUN: blood urea nitrogen and CRE: creatinine) were measured by using a blood biochemical autoanalyzer (7080, HITACHI, Japan). And the tumor tissues were weighed, and fixed in 4% neutral buffered formalin, processed routinely into paraffin, and sectioned at 4 μm. Then the sections were stained with hematoxylin and eosin (H&E) and TUNEL staining and finally examined by using an optical microscope (BX51, Olympus, Japan). Part of their organs were stained with H&E and examined as described above.

**Statistical analysis.**

Data analyses were conducted using the GraphPad Prism 5.0 software. Significance between every two groups was calculated by the Student’s t-test. *P < 0.05, **P < 0.01, ***P < 0.005.


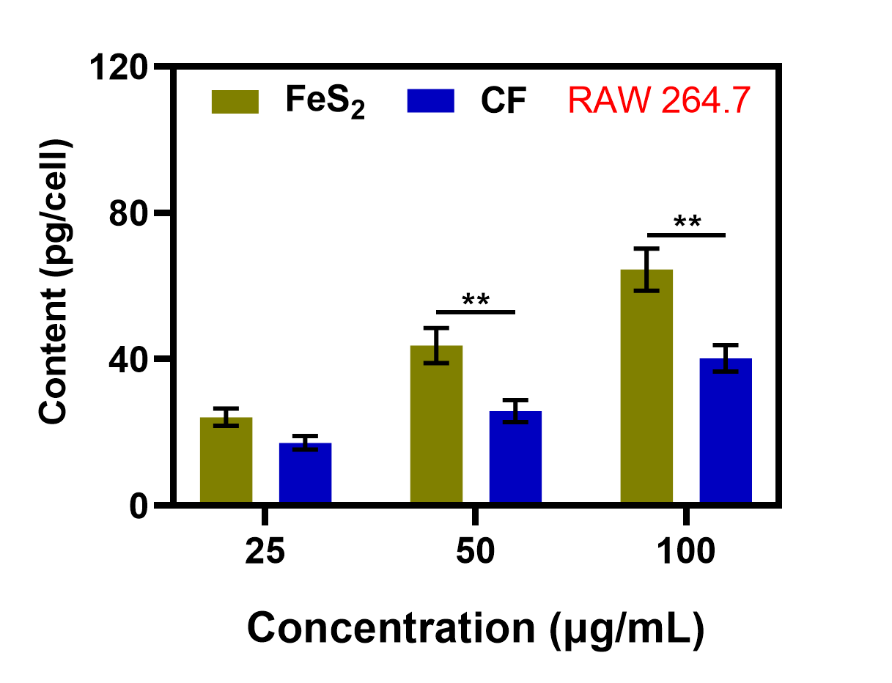


**Figure S1**. Nanoparticle uptake by RAW 264.7 cells at different incubated concentration (FeS_2_ dose of 25, 50 and 100 μg/mL). Data are presented as mean ± SD (n = 3).


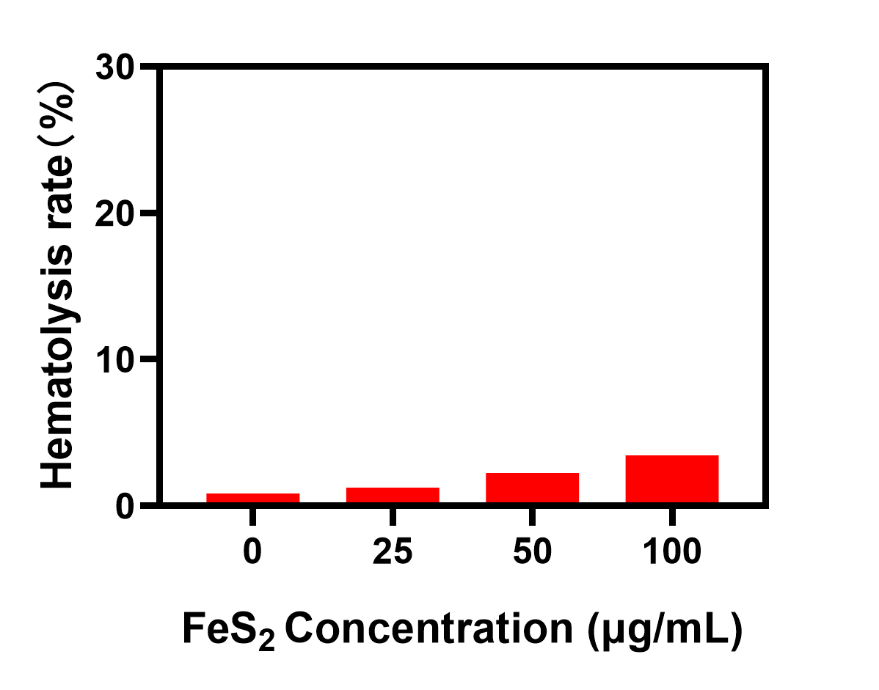


**Figure S2**. Hemolysis ratio of CF at different FeS_2_ concentrations.


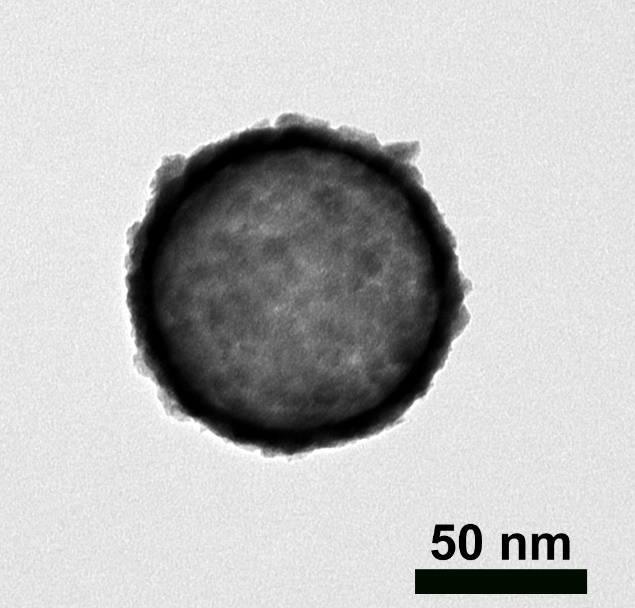
、

**Figure S3**.TEM image of MnO_2_.


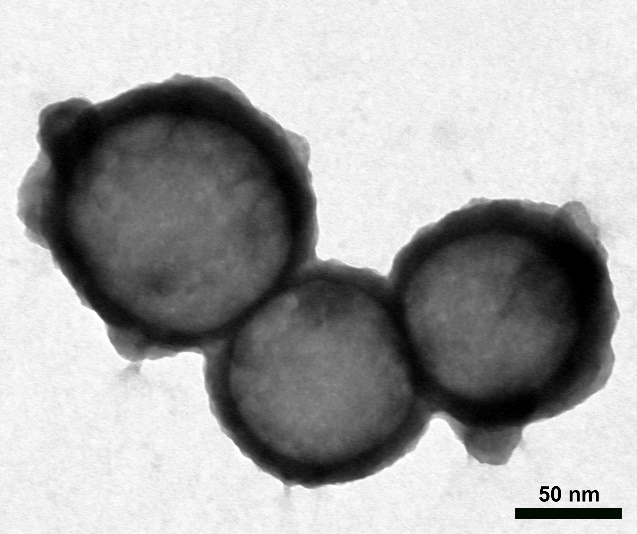


**Figure S4**.TEM image of CM.


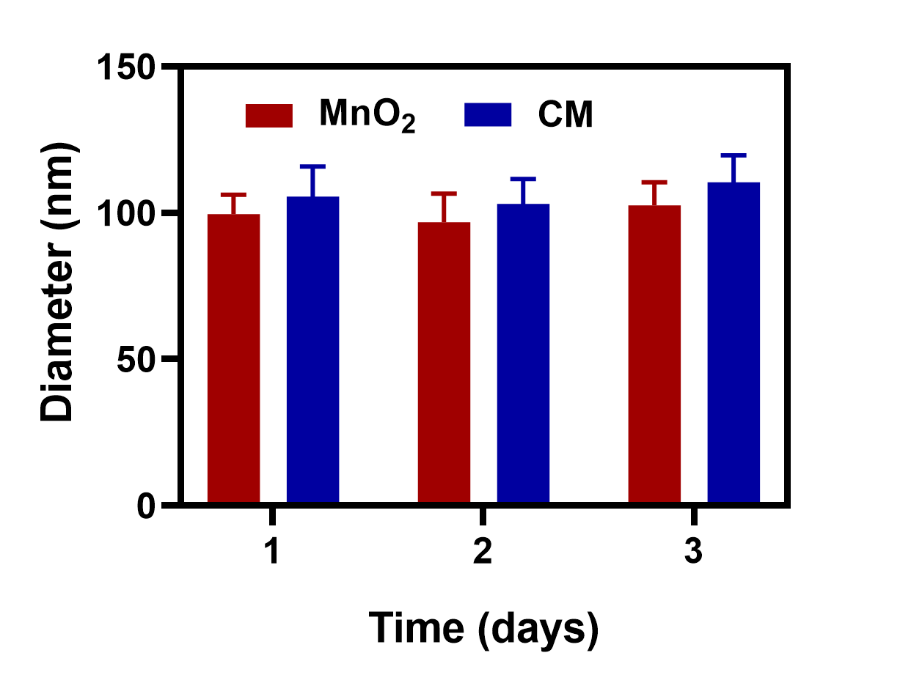


**Figure S5**. Statistical graph of the measured diameter of MnO_2_ and CM. Data are presented as mean ± SD (n = 3).


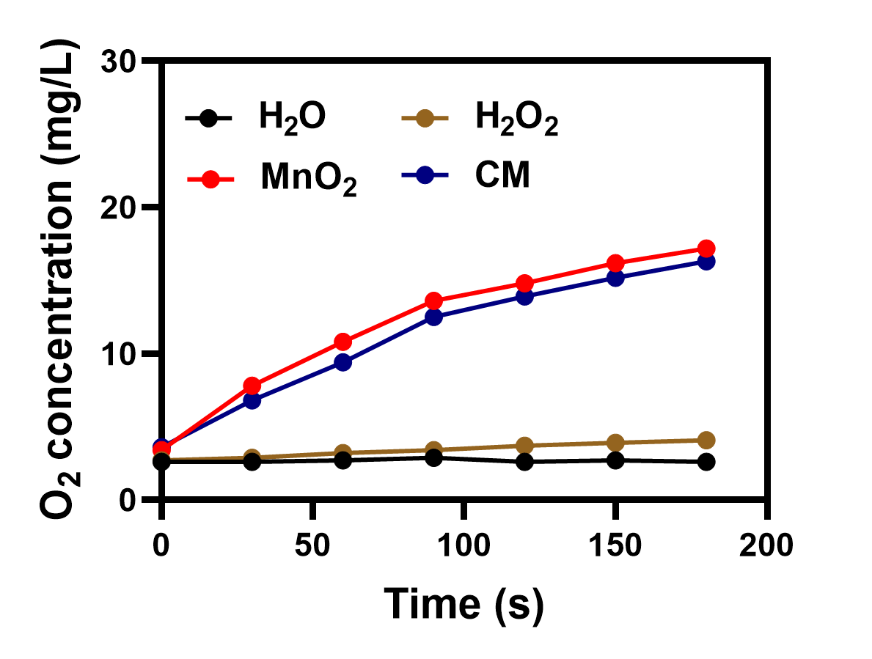


**Figure S6**. Oxygen generation under different conditions measured using a dissolved oxygen meter.


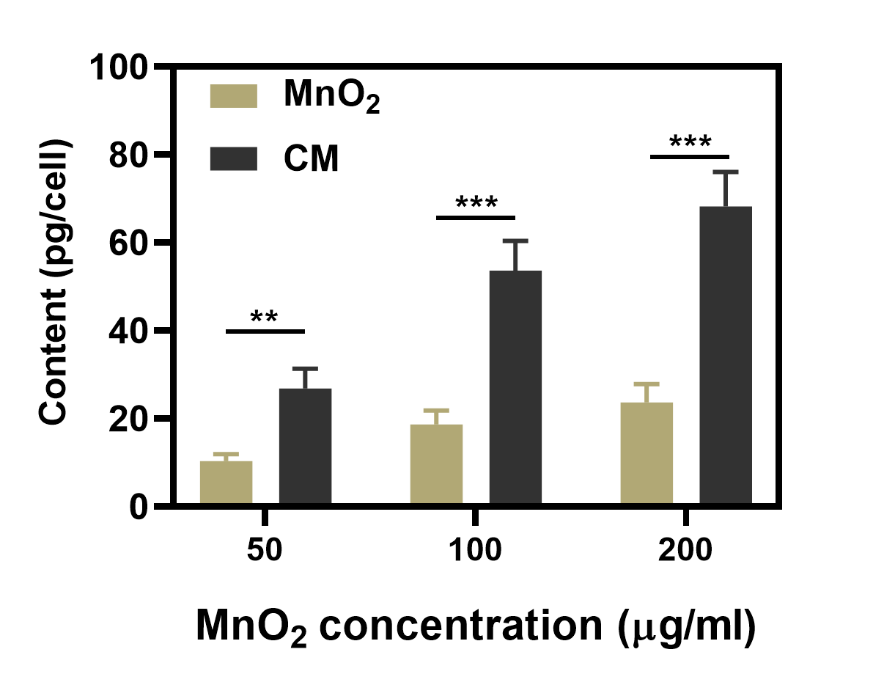


**Figure S7**. Nanoparticles uptake by 4T1 cells at different concentration. Data are presented as mean ± SD (n = 3).


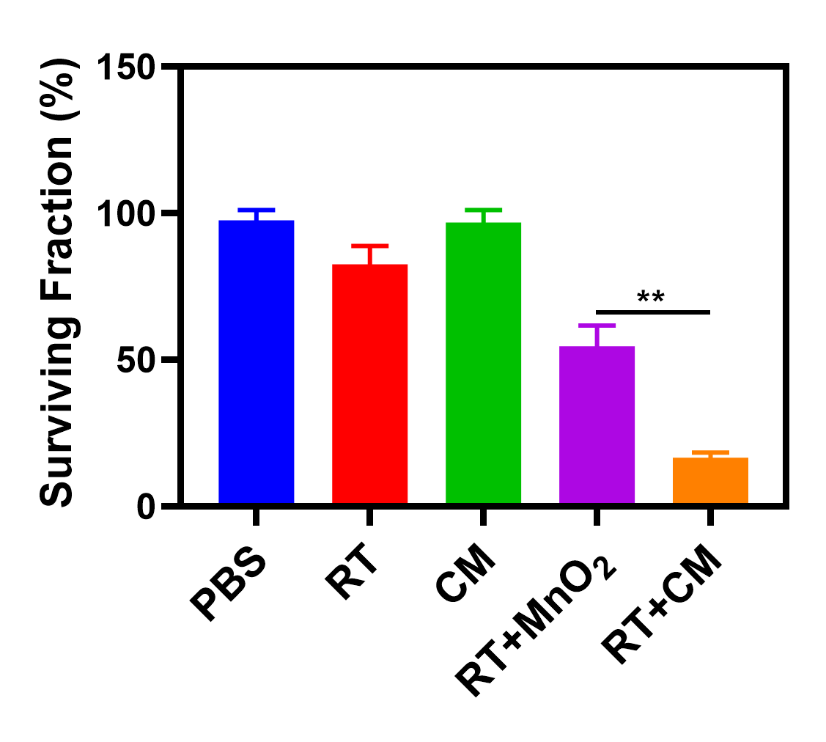


**Figure S8**. Colony formation assays were conducted using 4T1 cells with different treatment (n = 3).


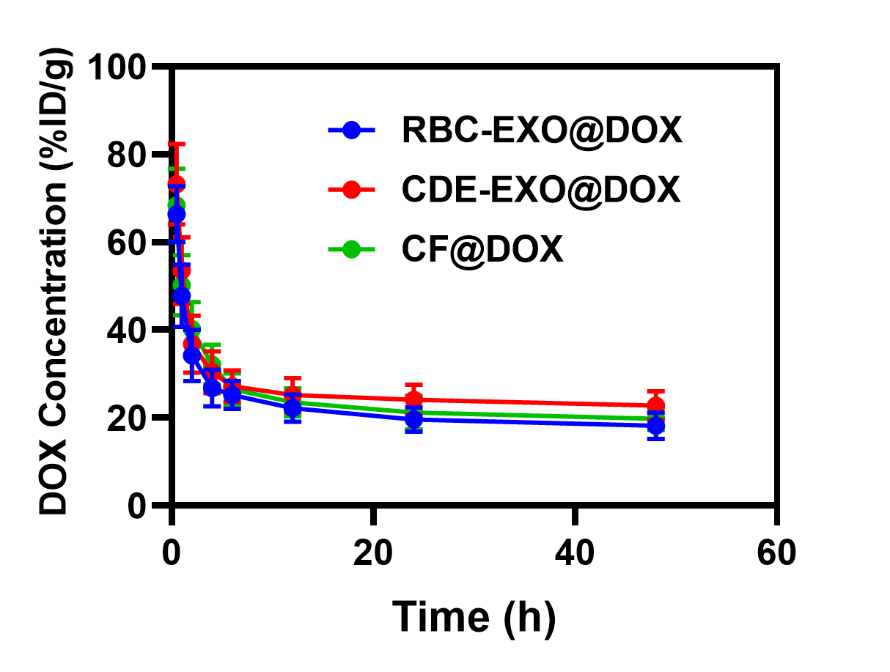


**Figure S9**. Pharmacokinetic behavior of RBC-EXO@DOX, CDE-EXO@DOX, and CF@DOX in mice following i.v. administration. Data are presented as mean ± SD (n = 3).


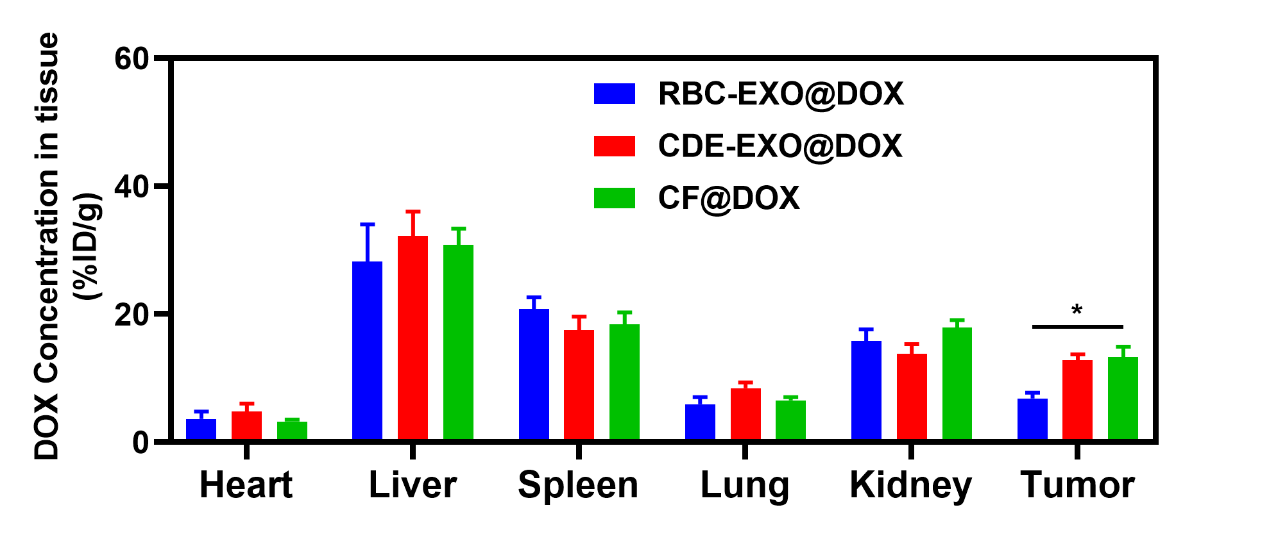


**Figure S10**. Quantitative analysis of DOX biodistribution in tissues and tumors of tumor-bearing mice injected with different formulations. Data are presented as mean ± SD (n = 3).

1. X. Meng, D. Li, L. Chen, H. He, Q. Wang, C. Hong, J. He, X. Gao, Y. Yang, B. Jiang, G. Nie, X. Yan, L. Gao and K. Fan: High-Performance Self-Cascade Pyrite Nanozymes for Apoptosis-Ferroptosis Synergistic Tumor Therapy. *ACS Nano* (2021) doi:10.1021/acsnano.1c01248

2. D. Zhu, M. Lyu, Q. Huang, M. Suo, Y. Liu, W. Jiang, Y. Duo and K. Fan: Stellate Plasmonic Exosomes for Penetrative Targeting Tumor NIR-II Thermo-Radiotherapy. *ACS Appl. Mater. Interfaces*, 12(33), 36928-36937 (2020) doi:10.1021/acsami.0c09969

3. D. M. Zhu, W. Xie, Y. S. Xiao, M. Suo, M. H. Zan, Q. Q. Liao, X. J. Hu, L. B. Chen, B. Chen, W. T. Wu, L. W. Ji, H. M. Huang, S. S. Guo, X. Z. Zhao, Q. Y. Liu and W. Liu: Erythrocyte membrane-coated gold nanocages for targeted photothermal and chemical cancer therapy. *Nanotechnology*, 29(8), 084002 (2018) doi:10.1088/1361-6528/aa9ca1

4. D. Zhu, M. Lyu, W. Jiang, M. Suo, Q. Huang and K. Li: A biomimetic nanozyme/camptothecin hybrid system for synergistically enhanced radiotherapy. *J Mater Chem B*, 8, 5312-5319 (2020) doi:10.1039/d0tb00676a
